# Supplementary material for: Perceived Risk and Intentions to Practice Health Protective Behaviors in a Mining-Impacted Region
Source: Int J Environ Res Public Health. 2020 Oct 28;17(21):7916. doi: 10.3390/ijerph17217916 (PMC7672644; doi:10.3390/ijerph17217916)
Supplement: Supplementary file 1 [file ijerph-17-07916-s001.zip › ijerph-974493 - supplementary tables final.docx]

**Table S1.** Exploratory factor analysis and descriptive statistics for perceived risk and behavioral intentions variables (*n* = 306).

|  | **Mean (SD)** | **^b^ Perceived Benefits** | | **Perceived Severity** | **Behavioral Intentions** | **Perceived Susceptibility** | **Self-Efficacy** | **Perceived Barriers** |
| --- | --- | --- | --- | --- | --- | --- | --- | --- |
| **Cronbach’s Alpha** | | **0.86** | | **0.84** | **0.83** | **0.82** | **0.87** | **0.81** |
| **Mean (SD)** | | | **3.9** | **3.0** | **3.8** | **2.2** | **3.1** | **3.4** |
|  |  |  | **(0.77)** | **(0.97)** | **(1.0)** | **(0.89)** | **(0.97)** | **(1.0)** |
| *Indicate to what extent you agree that completing the following actions are good for your health:* | | | | | | | | |
| Promptly removing dirt from your clothes, toys, pets, cars, and equipment after spending time outdoors. | 3.9 | 0.68 ^a^ | |  |  |  |  |  |
|  | (−0.89) |  | |  |  |  |  |  |
| Staying on designated trails while recreating in areas with lead contamination warning signs posted. | 3.9 | 0.79 | |  |  |  |  |  |
|  | (−0.95) |  | |  |  |  |  |  |
| Washing your hands with clean water or wipes before eating or drinking after recreating or working outdoors. | 4.2 | 0.74 | |  |  |  |  |  |
|  | (−0.76) |  | |  |  |  |  |  |
| Using a protective barrier such as a blanket when sitting on a sandy beach. | 3.8 | 0.72 | |  |  |  |  |  |
|  | (−0.95) |  | |  |  |  |  |  |
| Following the advice of a local public health official about ways to safely avoid lead contamination. | 3.9 | 0.8 | |  |  |  |  |  |
|  | (−0.87) |  | |  |  |  |  |  |
| I worry about lead contamination while spending time outdoors. | 2.6 |  | | 0.72 |  |  |  |  |
|  | (−1.18) |  | |  |  |  |  |  |
| It is worth my time to avoid lead contamination while spending time outdoors. | 3.3 |  | | 0.56 |  |  |  |  |
|  | (−1.16) |  | |  |  |  |  |  |
| I need more information about how to avoid lead contamination while spending time outdoors. ** | 2.9 |  | | 0.64 |  |  |  |  |
|  | (−1.28) |  | |  |  |  |  |  |
| I worry about lead contamination entering my home. | 2.6 |  | | 0.8 |  |  |  |  |
|  | (−1.26) |  | |  |  |  |  |  |
| It is worth my time to clean my home to prevent lead contamination. | 3.6 |  | | 0.59 |  |  |  |  |
|  | (−1.11) |  | |  |  |  |  |  |
| I need more information about how to prevent lead contamination from entering my home. ** | 3 |  | | 0.61 |  |  |  |  |
|  | (−1.23) |  | |  |  |  |  |  |
| *Consider your recreational and outdoor activities in your local area over the next 12 months. How likely is it that you will?* | | | | | | | | |
| Promptly remove dirt from your clothes, toys, pets, cars, and equipment after spending time outdoors? | 3.7 |  | |  | 0.55 |  |  |  |
|  | (1−1.33) |  | |  |  |  |  |  |
| Stay on designated trails while recreating in areas where lead contamination warning signs are posted? | 3.8 |  | |  | 0.69 |  |  |  |
|  | (−1.35) |  | |  |  |  |  |  |
| Wash your hands with clean water or wipes before eating or drinking after recreating or working outdoors? | 4.3 |  | |  | 0.56 |  |  |  |
|  | (−1.12) |  | |  |  |  |  |  |
| Use a protective barrier such as a blanket when sitting on a sandy beach? | 3.6 |  | |  | 0.63 |  |  |  |
|  | (−1.37) |  | |  |  |  |  |  |
| Follow the advice of a public health official about ways to avoid lead contamination while spending time outdoors? | 3.6 |  | |  | 0.83 |  |  |  |
|  | (−1.34) |  | |  |  |  |  |  |
| I have experienced health effects related to lead contamination. | 2 |  | |  |  | 0.91 |  |  |
|  | (−1.1) |  | |  |  |  |  |  |
| I feel I will experience health effects related to lead contamination at some time during my life. | 2.3 |  | |  |  | 0.94 |  |  |
|  | (−1.12) |  | |  |  |  |  |  |
| I am more likely than the average person to experience health effects from lead contamination. | 2.3 |  | |  |  | 0.66 |  |  |
|  | (−1.14) |  | |  |  |  |  |  |
| If it is my destiny to experience health effects related to lead contamination, there is nothing that I can do to prevent it. | 2.1 |  | |  |  | 0.4 |  |  |
|  | (−1.07) |  | |  |  |  |  |  |
| I know a lot about the health effects from lead contamination. | 3.2 |  | |  |  |  | 0.76 |  |
|  | (−1.04) |  | |  |  |  |  |  |
| I am better informed about the health effects of lead contamination than most people. | 3 |  | |  |  |  | 0.78 |  |
|  | (−1.03) |  | |  |  |  |  |  |
| I seek out information about lead contamination. ** | 2.7 |  | | 0.43 |  |  | 0.6 |  |
|  | (−1.96) |  | |  |  |  |  |  |
| I know how to prevent health effects from lead contamination. ** | 3.3 |  | |  |  |  | 0.45 | 0.36 |
|  | (−1.01) |  | |  |  |  |  |  |
| I know who to ask if I have questions about preventing health effects from lead contamination. | 3.5 |  | |  |  |  |  | 0.98 |
|  | (−1.04) |  | |  |  |  |  |  |
| I am aware of the available resources for preventing health effects of lead contamination. | 3.3 |  | |  |  |  |  | 0.63 |
|  | (−1.12) |  | |  |  |  |  |  |

Notes: Maximum likelihood extraction with direct oblimin rotation; 54% variance explained; Cut-off Eigenvalue = 1.00. ** Item not included in CFA or Cronbach’s Alphas ^a^ Values are factor loadings from the direct oblimin pattern matrix. Items with loadings below 0.30 were suppressed. ^b^ All variables are perceived variables derived from survey items.

**Table S2.** Structural equation model correlation matrix.

|  | **1** | **2** | **3** | **4** | **5** | **6** |
| --- | --- | --- | --- | --- | --- | --- |
| 1. Perceived Severity | 1 |  |  |  |  |  |
| 2. Perceived Susceptibility | 0.56 ** | 1 |  |  |  |  |
| 3. Perceived Benefits | 0.50 ** | 0.09 | 1 |  |  |  |
| 4. Perceived Barriers | −0.27 ** | −0.28 ** | 0.06 | 1 |  |  |
| 5. Self-Efficacy | −0.12 | −0.05 | 0.07 | 0.75 ** | 1 |  |
| 6. Behavioral Intentions | 0.51 ** | 0.17 ** | 0.75 ** | −0.03 | 0.06 | 1 |

Note: Correlation is significant at the 0.01 level (two-tailed). ** *p* < 0.01.
