# Supplementary material for: Perceived Risk and Intentions to Practice Health Protective Behaviors in a Mining-Impacted Region
Source: Int J Environ Res Public Health. 2020 Oct 28;17(21):7916. doi: 10.3390/ijerph17217916 (PMC7672644; doi:10.3390/ijerph17217916)
Supplement: Supplementary file 1 [file ijerph-17-07916-s001.zip › ijerph-974493 - supplementary file final.pdf]

Your Views of Health and Lead Contamination in North Idaho

University of Idaho
College of Natural Resources

Public Health
Prevent. Promote. Protect.
Panhandle Health District

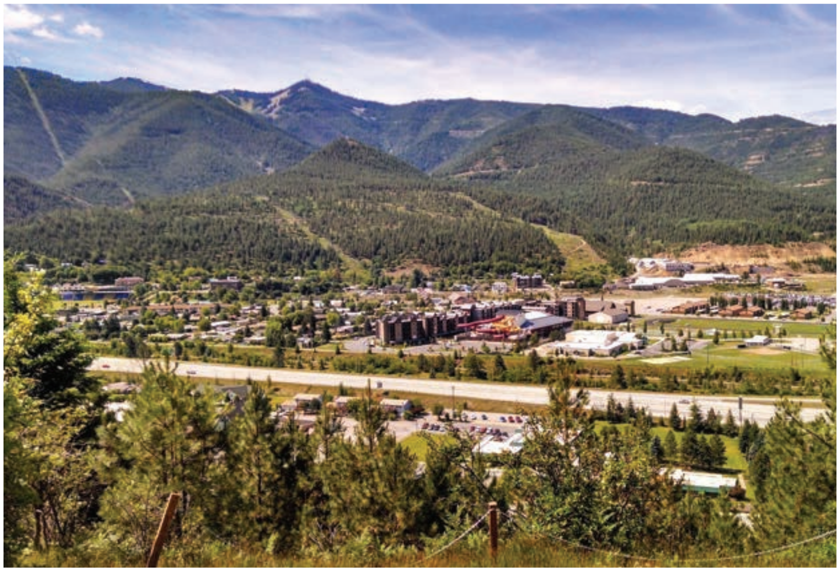

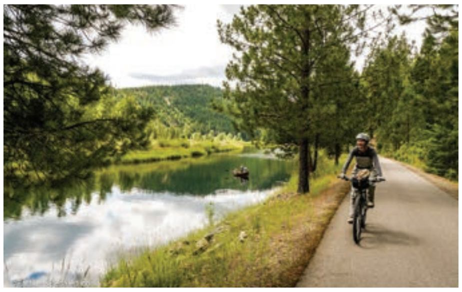

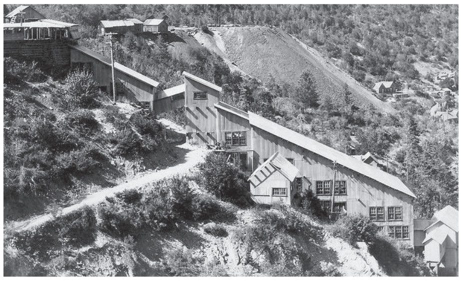

This survey is part of a study being conducted by researchers at the University of Idaho. We would like to understand how you view your health, as well as your opinions about lead contamination issues. We are contacting you as a resident of the Silver Valley in Shoshone County. Throughout this survey we will refer to this area as your "local area." Your answers are very important for us to understand different perspectives of people living in the Silver Valley. The survey will take about 15 minutes to complete. Thank you in advance for your participation!

Please answer the questions to the best of your ability. There are no right or wrong answers and you have the right to withdraw at any time. This study has been reviewed and meets protection assurance standards, including protection of privacy and confidentiality, by the University of Idaho's Institutional Review Board (IRB). This means that survey responses will not be linked to any identifying information.If you have questions regarding this study, please contact:

Courtney Cooper: ccooper@uidaho.edu
Dr. Chloe Wardropper: cwardropper@uidaho.edu
208-885-7911

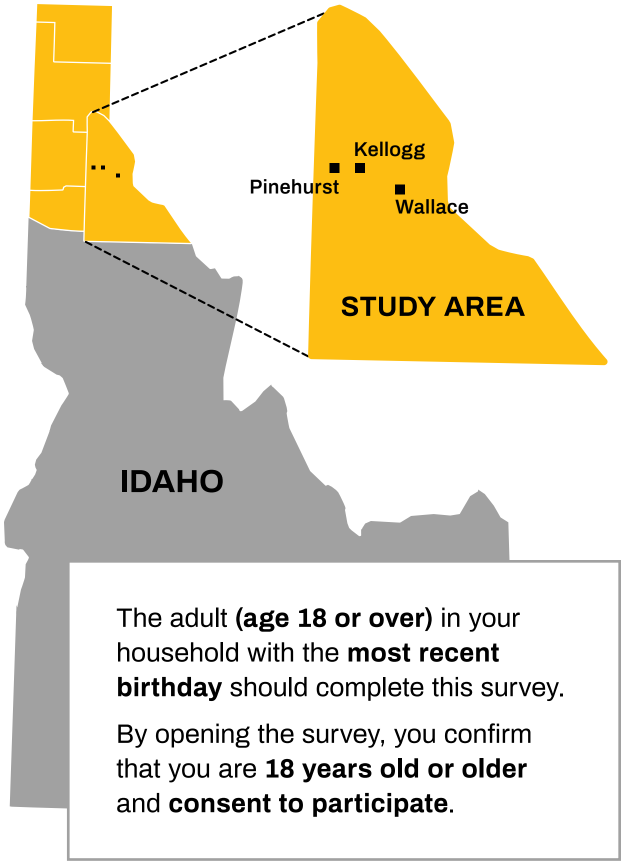

The adult **(age 18 or over)** in your household with the **most recent birthday** should complete this survey.

By opening the survey, you confirm that you are **18 years old or older** and **consent to participate**.

Image Citations | left: City of Kellogg; top right: Path Less Pedaled; bottom right: University of Idaho Digital Archive

INSTRUCTIONS: Please answer every question by marking one box or filling in the blank. If you are unsure about how to answer, please give the best answer you can.

1. How many total years you have lived in north Idaho? Please include **all years of residence**, even if they were not consecutive and round to the nearest **whole number**.

☐ years

The next questions ask for your views about your health. This information will keep track of how you feel and how well you are able to do your usual activities.

2. **In general, would you say your health is...?**

☐ Excellent ☐ Very Good ☐ Good ☐ Fair ☐ Poor

3. **The following items are about activities you might do during a typical day. Does <u>your health now limit you</u> in these activities? If so, how much?**

	Yes, Limited A Lot	Yes, Limited A Little	No, Not Limited At All
a. **Moderate activities**, such as moving a table, pushing a vacuum cleaner, bowling, or playing golf	☐	☐	☐
b. Climbing **several** flights of stairs	☐	☐	☐

4. **During the <u>past 4 weeks</u>, have you had any of the following problems with your work or other daily activities <u>as a result of your physical health</u>?**

	Yes	No
a. **Accomplished less** than you would like	☐	☐
b. Were limited in the **kind** of work or other activities	☐	☐

5. **During the <u>past 4 weeks</u>, have you had any of the following problems with your work or other regular daily activities <u>as a result of any emotional problems</u> such as feeling depressed or anxious?**

	Yes	No
a. **Accomplished less** than you would like	☐	☐
b. Didn't do work or activities as **carefully** as usual	☐	☐

6. During the past 4 weeks, how much did pain interfere with your normal work (including both outside the home and housework)?

☐ Not at all ☐ A little bit ☐ Moderately ☐ Quite a bit ☐ Extremely

7. These questions are about how you feel and how things have been with you during the past 4 weeks. For each question, please give the one answer that comes closest to the way you have been feeling.

How much of the time during the past 4 weeks:	All of the Time	Most of the Time	A Good Bit of the Time	Some of the Time	A Little of the Time	None of the Time
a. Have you felt calm and peaceful?	☐	☐	☐	☐	☐	☐
b. Did you have a lot of energy?	☐	☐	☐	☐	☐	☐
c. Have you felt downhearted or blue?	☐	☐	☐	☐	☐	☐

8. During the past 4 weeks, how much of the time has your physical health or emotional problems interfered with your social activities (like visiting with friends, relatives, etc.)?

All of the Time	Most of the Time	Some of the Time	A Little of the Time	None of the Time
☐	☐	☐	☐	☐

The next few questions relate to how frequently you engage in different activities. These include activities associated with potential lead contamination issues in your local area.

9. Consider your activities in your local area over the PAST 12 months.

How often have you:	Never	Rarely	Sometimes	Often	Very Often
a. Participated in outdoor work or recreation activities?	☐	☐	☐	☐	☐
b. Thought about lead contamination?	☐	☐	☐	☐	☐
c. Read or heard about lead contamination?	☐	☐	☐	☐	☐
d. Attended a public meeting or event about lead contamination?	☐	☐	☐	☐	☐

10. Please indicate which meetings or events you have attended in your local area in the PAST 12 months.

☐ Basin Environmental Improvement Commission Meeting ☐ Citizens Coordinating Council Meeting

☐ Earth Day Fair Coeur d'Alene ☐ Our Gem Symposium ☐ Shoshone Medical Center's Kids Health & Safety Fair

☐ Silver Valley Community Resource Center Event ☐ Spokane River Forum

☐ Other → Please list:

11. Consider your recreational and outdoor activities in <u>your local area in the PAST 12 months</u>.

**How often have you:**	Never	Rarely	Sometimes	Often	Very Often
a. Promptly removed dirt from your clothes, toys, pets, cars, and equipment after spending time outdoors?	☐	☐	☐	☐	☐
b. Stayed on designated trails while recreating in areas where lead contamination warning signs are posted?	☐	☐	☐	☐	☐
c. Recreated in or near the South Fork of the Coeur d'Alene River?	☐	☐	☐	☐	☐
d. Washed your hands with clean water or wipes before eating or drinking after recreating or working outdoors?	☐	☐	☐	☐	☐
e. Used a protective barrier such as a blanket when sitting on a sandy beach?	☐	☐	☐	☐	☐
f. Followed the advice of a public health official about ways to avoid lead contamination while spending time outdoors?	☐	☐	☐	☐	☐

12. Consider your recreational and outdoor activities in <u>your local area over the NEXT 12 months</u>.

**How likely is it that you will:**	Very unlikely	Unlikely	Neutral	Likely	Very Likely	Does not apply
a. Promptly remove dirt from your clothes, toys, pets, cars, and equipment after spending time outdoors?	☐	☐	☐	☐	☐	☐
b. Stay on designated trails while recreating in areas with lead contamination warning signs posted?	☐	☐	☐	☐	☐	☐
c. Recreate in or near the South Fork of the Coeur d'Alene River?	☐	☐	☐	☐	☐	☐
d. Wash your hands with clean water or wipes before eating or drinking after recreating or working outdoors?	☐	☐	☐	☐	☐	☐
e. Use a protective barrier such as a blanket when sitting on a sandy beach?	☐	☐	☐	☐	☐	☐
f. Follow the advice of a public health official about ways to avoid lead contamination while spending time outdoors?	☐	☐	☐	☐	☐	☐

The next questions ask about your views of lead contamination issues in your local area and household.

13. Consider your level of concern about environmental issues.

How concerned are you about:	Not at all concerned	Slightly concerned	Somewhat concerned	Moderately concerned	Extremely concerned
a. Global human health issues?	☐	☐	☐	☐	☐
b. Human health issues in your local area?	☐	☐	☐	☐	☐
c. Health issues in your household?	☐	☐	☐	☐	☐
d. Human health issues related to lead contamination in your local area?	☐	☐	☐	☐	☐
e. Health issues related to lead contamination in your household?	☐	☐	☐	☐	☐

14. Indicate how much you agree with these statements regarding possible lead contamination in your local area.

	Strongly Disagree	Disagree	Neither	Agree	Strongly Agree
a. I worry about lead contamination while spending time outdoors.	☐	☐	☐	☐	☐
b. It is worth my time to avoid lead contamination while spending time outdoors.	☐	☐	☐	☐	☐
c. I can avoid lead contamination while spending time outdoors.	☐	☐	☐	☐	☐
d. I need more information about how to avoid lead contamination while spending time outdoors.	☐	☐	☐	☐	☐
e. Avoiding lead contamination while spending time outdoors is inconvenient.	☐	☐	☐	☐	☐

15. Indicate how much you agree with these statements regarding possible lead contamination around your home.

	Strongly Disagree	Disagree	Neither	Agree	Strongly Agree
a. I worry about lead contamination entering my home.	☐	☐	☐	☐	☐
b. It is worth my time to clean my home to prevent lead contamination.	☐	☐	☐	☐	☐
c. I can prevent lead contamination from entering my home.	☐	☐	☐	☐	☐
d. I need more information about how to prevent lead contamination from entering my home.	☐	☐	☐	☐	☐
e. Preventing lead contamination from entering my home is inconvenient.	☐	☐	☐	☐	☐

The next questions ask about your views of the relationship between health and lead contamination in your local area.

16. Indicate how much you agree with these statements regarding possible lead contamination in your local area.

	Strongly Disagree	Disagree	Neither	Agree	Strongly Agree
a. **Clear water** in the rivers and streams indicates that the water is safe from lead contamination.	☐	☐	☐	☐	☐
b. **Healthy vegetation** along the banks of the rivers and streams indicates that the soil is safe from lead contamination.	☐	☐	☐	☐	☐

17. Indicate how much you agree with these statements regarding your views of how public money should be spent on issues related to lead contamination in your local area.

	Strongly Disagree	Disagree	Neither	Agree	Strongly Agree
a. Public money should be used to limit lead contamination in **rivers, streams, and beaches**.	☐	☐	☐	☐	☐
b. Public money should be used to limit lead contamination in **cities and towns**.	☐	☐	☐	☐	☐
c. Public money should be used to **educate people** about lead contamination.	☐	☐	☐	☐	☐

18. How much do you trust the following sources to provide you with accurate information about health and lead contamination in your local area?

	Not Much	Little	Somewhat	Much	A Great Deal
a. Community leaders	☐	☐	☐	☐	☐
b. Family members	☐	☐	☐	☐	☐
c. Friends	☐	☐	☐	☐	☐
d. Scientists	☐	☐	☐	☐	☐
e. My public health district	☐	☐	☐	☐	☐
f. Doctors	☐	☐	☐	☐	☐

The next few questions ask about your views of the health effects of lead contamination issues in your local area.

19. Indicate how much you agree with these statements regarding how <u>lead contamination in your local area affects your health</u>.

	Strongly Disagree	Disagree	Neither	Agree	Strongly Agree
a. I have experienced health effects related to lead contamination.	☐	☐	☐	☐	☐
b. I feel I will experience health effects related to lead contamination at some time during my life.	☐	☐	☐	☐	☐
c. I am more likely than the average person to experience health effects from lead contamination.	☐	☐	☐	☐	☐
d. If it is my destiny to experience health effects related to lead contamination, there is nothing that I can do to prevent it.	☐	☐	☐	☐	☐

20. Indicate how much you agree with these statements regarding how <u>you address the health effects of lead contamination in your local area</u>.

	Strongly Disagree	Disagree	Neither	Agree	Strongly Agree
a. I know how to prevent health effects from lead contamination.	☐	☐	☐	☐	☐
b. I know who to ask if I have questions about preventing health effects from lead contamination.	☐	☐	☐	☐	☐
c. I am aware of the available resources for preventing health effects of lead contamination.	☐	☐	☐	☐	☐

21. Indicate how much you agree with these statements <u>regarding your awareness of lead contamination in your local area</u>.

	Strongly Disagree	Disagree	Neither	Agree	Strongly Agree
a. I know a lot about the health effects of lead contamination.	☐	☐	☐	☐	☐
b. I am better informed about the health effects of lead contamination than most people.	☐	☐	☐	☐	☐
c. I seek out information about lead contamination.	☐	☐	☐	☐	☐
d. A small amount of lead in the human body is harmful.	☐	☐	☐	☐	☐

22. Indicate to what extent you agree that completing the following actions are good for your health. Think about each statement in relation to activities in your local area.

	Strongly Disagree	Disagree	Neither	Agree	Strongly Agree
a. Promptly removing dirt from your clothes, toys, pets, cars, and equipment after spending time outdoors.	☐	☐	☐	☐	☐
b. Staying on designated trails while recreating in areas with lead contamination warning signs posted.	☐	☐	☐	☐	☐
c. Washing your hands with clean water or wipes before eating or drinking after recreating or working outdoors.	☐	☐	☐	☐	☐
d. Using a protective barrier such as a blanket when sitting on a sandy beach.	☐	☐	☐	☐	☐
e. Following the advice of a local public health official about ways to safely avoid lead contamination.	☐	☐	☐	☐	☐

23. Identify the level of health risk associated with lead contamination in your local area for the following.

	No risk	Low risk	Moderate risk	High risk	Very high risk	Don't Know
a. Level of immediate health risk associated with lead contamination for me.	☐	☐	☐	☐	☐	☐
b. Level of long-term health risk associated with lead contamination for me.	☐	☐	☐	☐	☐	☐
c. Level of immediate health risk associated with lead contamination for others in my local area.	☐	☐	☐	☐	☐	☐
d. Level of long-term health risk associated with lead contamination for others in my local area.	☐	☐	☐	☐	☐	☐

Finally, we have a few questions about you and your household.

24. What is your gender?

- ☐ Male
- ☐ Female
- ☐ Prefer not to say
- ☐ Other

25. What year were you born?

☐☐☐☐ Year

26. What year was your home built in?

☐☐☐☐ Year

27. If you have children, what is the age of your youngest child? *Please check "N/A" if you do not have children.*

☐ Age ☐ N/A

28. Has a member of your household ever worked in a mining-related job in your local area?

- ☐ Yes
- ☐ No
- ☐ I don't know

29. Which of the following racial or ethnic groups do you most closely identify with?

- ☐ American Indian or Alaskan Native
- ☐ Asian
- ☐ Black or African American
- ☐ White
- ☐ Biracial or multiracial
- ☐ Other → Please share:

30. What is the highest level of formal education you have completed or the highest degree you have received?

- ☐ Less than high school degree
- ☐ High school graduate (high school diploma or equivalent including GED)
- ☐ Some college but no degree
- ☐ College degree (2 or 4 year)
- ☐ Advanced degree (Masters, Ph.D., J.D., M.D.)

31. What is your occupational status?

- ☐ Homemaker
- ☐ Retired
- ☐ Student
- ☐ Unemployed
- ☐ Working part-time
- ☐ Working full-time
- ☐ Other → Please specify:

32. What is your total annual household income, before taxes? Please give your best guess.

- ☐ Less than $20,000
- ☐ $20,000 to $49,999
- ☐ $50,000 to $79,999
- ☐ $80,000 to $99,999
- ☐ $120,000 or more

Thank you for taking the time to fill out our survey! We really appreciate your participation. If you have any comments on the survey or about lead contamination or health in your local area, please comment below.

You are now finished with the survey. Please follow the instructions provided to return the survey.
